# On the Behavior of Honeycomb, Grid and Triangular PLA Structures under Symmetric and Asymmetric Bending

**DOI:** 10.3390/mi14010120

**Published:** 2022-12-31

**Authors:** Vasile Cojocaru, Doina Frunzaverde, Calin-Octavian Miclosina

**Affiliations:** Department of Engineering Science, Babeș-Bolyai University, Traian Vuia Square 1-4, 320085 Reșița, Romania

**Keywords:** additive manufacturing, fused filament fabrication (FFF), polylactic acid (PLA), honeycomb, grid, triangular, symmetric bending, asymmetric bending

## Abstract

Additive manufacturing technologies enable the production of components with lightweight cores, by means of infills with various patterns and densities. Together with reduced mass and material consumption, infill geometries must ensure that strength and stiffness conditions are fulfilled. For the proper correlation of the infill type with the loading case of the part, the mechanical behavior of the infill along all three principal axes of inertia has to be known. In this paper, the behavior in symmetric and asymmetric bending of three infill geometries, commonly used in 3D printing processes (honeycomb, grid and triangles) is analyzed. The variations of deflections as a function of force orientation are presented, showing that honeycomb and triangular structures exhibit similar behaviors along the *Y* and *Z* principal axes of inertia. Furthermore, the displacements obtained for the three types of structures are compared, in relation to the consumed volume of material. The larger displacements of the grid structure compared to the honeycomb and triangular structures are highlighted.

## 1. Introduction

Additive manufacturing technologies have developed intensively in recent decades. Specific to these technologies is the building of parts layer by layer, allowing the core of the parts to be generated with different infill geometries (alternative to full core parts). A decrease in mass and material consumption is achieved using an infill pattern. Furthermore, the infill patterns should be selected in relation to the loads applied to the part, aiming to optimize the stress distribution and static/dynamic behavior [1,2,3,4].

Infill geometries are defined by two main parameters: the infill pattern and the infill density [5]. The various slicer software used in FFF (fused filament fabrication) technology have predefined various infill patterns, the number of which has been increasing in recent years. Engineers can also build optimized infill geometries directly in computer-aided design (CAD) software according to the stress and strain states of the parts. Several categories of infill geometries can be defined:

- Infill geometries defined by a 2D model, identical for two successive layers (the result is a 2.5D structure with prismatic cells oriented along the *Z* axis of the printer);
- Infill geometries defined by 3D models (the infill pattern is defined by several successive layers with different configurations; this pattern is repeated after a specific number of layers; the cells can be open or closed) [6,7];
- Lattice-type infill geometries [8];
- Infill geometries with variable infill, changed from layer to layer [9] or changed between specified areas in the part [10,11,12];
- Infill geometries with variable structure resulting from topological optimization.

**Figure 1:**
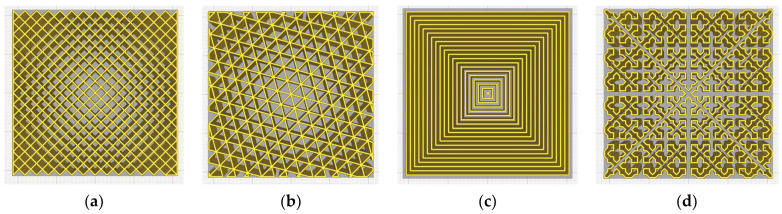
The top views of a prismatic volume (40 × 40 × 10 mm^3^) with various infill patterns defined by 2D models: (**a**) grid, (**b**) triangles, (**c**) concentric, (**d**) cross.

Figure 1 shows examples of infill geometries defined by 2D models. The four examples (grid, triangles, concentric, cross) were generated with an infill density of 40%, for a prismatic volume 40 × 40 × 10 mm^3^, using the Ultimaker Cura 5.0.0 slicer software (Ultimaker B.V., Utrecht, Netherlands). For a better display, the infill geometries were generated without top/bottom layers and without peripheral contours (without shell). 

**Figure 2:**
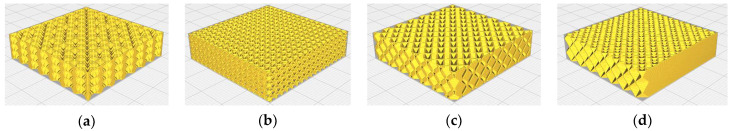
The perspective views of a prismatic volume (40 × 40 × 10 mm^3^) with various infill patterns defined by 3D models: (**a**) cross 3D, (**b**) gyroid, (**c**) quarter cubic, (**d**) octet subdivision.

Figure 2 shows four examples of infill geometries defined by 3D models, generated under the same conditions presented above.

**Figure 3:**
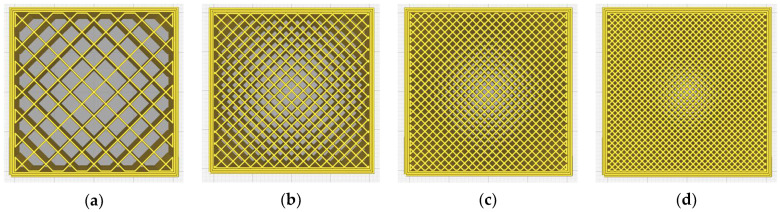
The top views of a prismatic volume (40 × 40 × 10 mm^3^) with grid infill at various densities: (**a**) 20%, (**b**) 40%, (**c**) 60%, (**d**) 80%.

The density of the infill indicates the percentage of the inner volume that is occupied by material (100% infill density indicates a solid volume; 0% infill density indicates a hollow part). Figure 3 shows a 40 × 40 × 10 mm^3^ prismatic volume with grid infill at four different densities: 20%, 40%, 60% and 80%. The infill was generated by the Ultimaker Cura 5.0.0 slicer software, using three perimeter contours and without upper and lower layers.

**Figure 4:**
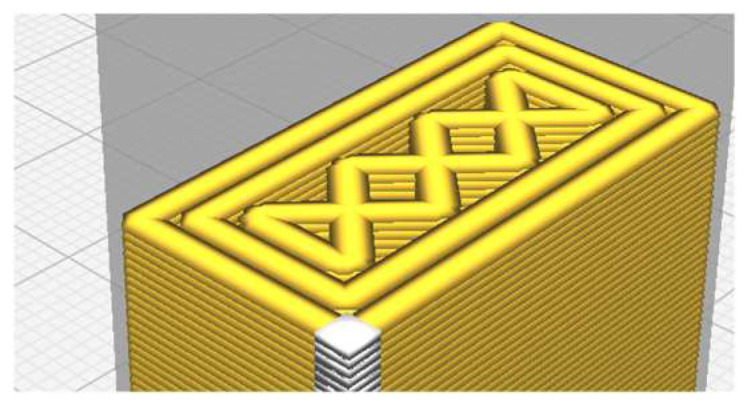
Cross-section of an ASTM D638-14 type IV specimen with 90% grid infill, two wall lines and ZY build orientation.

The peripheral contours, the lower layers and the upper layers constitute a shell that closes the filling volume and gives the shape of the piece. The ratio of the shell material volume to the infill material volume increases under certain conditions: small parts; low infill densities; high number of top/bottom layers and peripheral contours. In such cases, the contribution of the shell to the load bearing capacity of the piece increases as well [13]. An eloquent example in this regard is a tensile specimen with a small cross-section. For instance, the area occupied by the infill of an ASTM D638-14 type IV tensile specimen [14] with a 3.2 × 6 mm^2^ cross-section, printed in the ZX or ZY build orientation [15], with two peripheral layers of 0.4 mm (Figure 4), represents only 37% of the cross-section. To conclude, for an accurate analysis of the results obtained from mechanical tests on specimens with small cross-sections, the following parameters should also be considered: the number and the thickness of the lower and upper layers, the wall line number, and the wall thickness.

In the fused filament fabrication (FFF) technology, polymeric materials (polylactic acid—PLA, acrylonitrile butadiene styrene—ABS, polyethylene terephthalate—PET, etc.) and composite materials are used. For the components made by FFF printing, the mechanical properties are influenced by several process parameters, but also by pre-process conditions (filament state) and post-process conditions (storage conditions, post-process treatments, working environment) [16,17]. The orientation of the part on the printer build plate and the type of raster can determine the anisotropic behavior of the material (even if the material is isotropic in conventional manufacturing). Differences are observed mainly between the behavior on the two axes of the printer build plate (*X* and *Y*, according to ISO/ASTM 52921:2013) and the *Z* axis on which successive layers are built [16]. The pattern and the density of infill can increase the anisotropic behavior. Under these conditions, it is necessary to carry out the characterization of the mechanical behavior of components with different types of infill following each of the three principal axes of inertia.

Research carried out so far on polymeric materials printed with different infills has mainly focused on the analysis of tensile, compressive or bending behavior. A synthesis of the literature is presented below, including only papers in which specimens are printed from polylactic acid using the FFF technology.

Doboș et al. [18] analyze the tensile behavior for four types of infill (rectilinear, Hilbert curve, concentric and honeycomb) with 40–80% densities. The force–elongation curves show that at 80% density the highest breaking force is obtained for honeycomb infill, while at 40% and 60% densities the highest breaking force is obtained for concentric infill. 

Guan [19] compares the tensile behavior of honeycomb, triangular and rectilinear infill specimens and shows that both UTS and Young’s modulus (E) are higher for honeycomb and triangular infill.

Gonzalez-Rebanque et al. [20] analyze the tensile behavior of PLA specimens made with rectilinear, triangle and honeycomb infill and show that at 80% infill density the highest value of UTS (ultimate tensile strength) is obtained for rectilinear infill specimens, and at 50% and 30% densities the highest UTS value is obtained for honeycomb infill.

In [21] the tensile behavior of six types of infill is analyzed: hexagonal, triangular, square, diamond, diamond angle and square angle. These infill structures were disposed only in the calibrated area of the ASTM D638 Type I specimen directly by CAD modeling. No top layers were defined, but peripheral contours were used. The stress–strain curves show that the highest tensile strengths were obtained for the square and triangle structures and the lowest tensile strength was obtained for the diamond structure. 

Comparative analysis of tensile behavior for the ASTM D638-type I specimens with three peripheral contours [22] indicates that at 75% infill density, concentric-type structures have higher UTS compared to grid and tri-hexagonal structures.

The comparison of the tensile behavior of ISO 527-2 [23] specimens made with seven infill patterns, 60% infill density and two peripheral contours shows close values of the mechanical strength [24]. Analyzing the specimens, it can be concluded that the peripheral contours determine the mechanical behavior while the infill influence is low.

Research by Alafaghani et al. [25] indicates that the UTS obtained for ASTM D638 type IV specimens made with hexagonal infill and diamond infill is slightly higher than the UTS obtained for specimens with linear infill. The small differences obtained can be correlated with the small dimensions of the specimen cross-section (6 × 3.5 mm^2^) and the influence of the shell walls.

Tensile tests performed on ASTM D638 specimens [26] showed that the UTS is higher for grid infill compared to triangle and gyroid. This conclusion was obtained for all three infill densities analyzed (60%, 75%, 90%). The authors explain this behavior by the alignment of the grid infill with the axis of the tensile specimen.

Pernet and co-authors [27] test 14 types of infill in compression and plot the ratios of maximum compressive force to weight of specimens. The specimens used are cylindrical with a diameter of 12.7 mm and a length of 25.4 mm, and have three peripheral contours. The highest force/mass ratios are obtained for grid and cross infills.

In [28] the compression behavior of prismatic specimens (12.7 × 12.7 × 25.4 mm^3^) with six types of infill (octagram spiral, Hilbert curve, rectilinear, line, Archimedean curve and honeycomb) and four infill densities (ranging from 20% to 80%) are analyzed. It is shown that the Hilbert curve pattern specimens have the highest compressive strength (approximately double compared to the other infills). 

Analyzing four types of infill (triangle, grid, quarter-cubic and tri-hexagon) in low-velocity impact tests and a compression test, Aloyaydi et al. [29] show that the grid type infill has the highest compressive strength and the triangle type infill has the highest absorbed energy in impact tests.

Birosz et al. [30] compare the bending behavior for three types of infill: grid, honeycomb and gyroid. The specimens with square cross-section of 10 × 10 mm^2^ were made with shells of constant thickness—0.8 mm (the section occupied by the infill is 8.4 × 8.4 mm^2^). The tests were performed with the force orientated along two axes, *Y* and *Z*. The results obtained by the authors highlight the higher breaking force of specimens with hexagonal and gyroid infill. It is noted that the breaking force and modulus of elasticity obtained along the X-axis are close to the breaking force and modulus of elasticity obtained along the Z-axis (this is evidenced for all three types of infill). 

Honeycomb infill specimens (10×4 mm^2^ cross-section) show higher modulus of elasticity compared to linear and rectilinear infill specimens in four-point bending tests [31].

In [32] the bending properties of grid and honeycomb infills made at three different densities (10%, 25% and 50%) are analyzed. For the 50% density the honeycomb infill shows flexural strength higher by approx. 13% compared to the grid infill. The ISO 178:2011 [33] type specimens (10 × 4 mm^2^ section) are made with three peripheral contours. At low infill densities, the influence of the shell on the bending behavior can be higher than the influence of the infill. 

In [34] the tensile and bending behavior of ASTM D638-14 [14] and ASTM D790-10 [35] specimens with rectilinear, concentric, honeycomb and Hilbert-curve infill are analyzed. No details are given on printing parameters, number of peripheral contours or number of top layers. In both bending and tensile tests, the highest mechanical strengths were obtained for the rectilinear and honeycomb infills. 

The analysis of the impact behavior (Izod test) for 12 infill patterns at different infill densities reveals that the linear, zig-zag and concentric infills exhibit the highest energy absorbed on impact, at both 50% and 75% density [36].

Bonada et al. [37] analyze the type of infill correlated with the build orientation of the specimens and the type of the raster. It should be noted that the build orientation causes significant differences in infill orientation and specimen mass. Thus, at XY and YX orientations the infill is built on the thickness of the specimen, at YZ and XZ orientations the infill is built on the width of the specimen, and at ZY and ZX orientations the infill is built on the length of the specimen. 

Most of the research reviewed was conducted on standardized specimens. The reduced thickness of these specimens leads to a low ratio of the infill material volume to the shell material volume. Under these conditions, the relevance of analyses of the mechanical behavior of different infill patterns is low. Furthermore, the analysis of the variation of the mechanical properties of the cellular structures in relation to the orientation of the force to the main axes of inertia is less studied in the literature.

The objective of the current research was to analyze the symmetric and asymmetric bending behavior for three types of infill structures widely used in additive manufacturing: honeycomb, grid and triangular. In particular, it aimed to determine the variation of the deflections as a function of the orientation of the force to the principal axes of inertia *Y* and *Z*. Furthermore, considering the volume of each type of structure, a comparative analysis of the behavior of the three structures was also carried out. The characterization of asymmetric bending behavior of infill structures is of major importance for mechanical components subjected to loads with variable direction (e.g., turbine blades, propellers).

## 2. Materials and Methods

**Figure 5:**
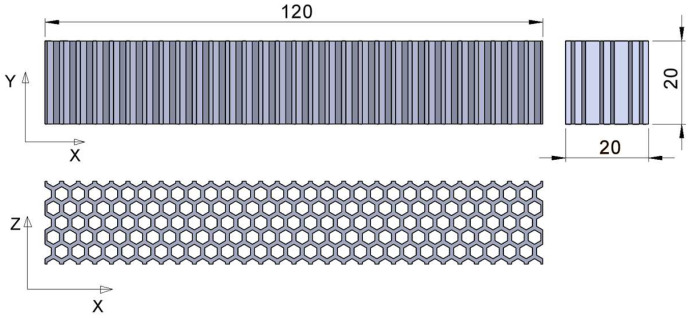
The specimens’ overall dimensions—illustration for the hexagonal structure.

The geometries of the three types of structures analyzed in this paper (honeycomb, grid and triangles) were modelled in the SolidWorks 2020-2021 software (Dassault Systèmes, Vélizy-Villacoublay, France). The structures were fitted into a prismatic volume of 120 mm length and 20 × 20 mm^2^ cross-section (Figure 5). A square cross-section was chosen so that a comparative analysis of *Z*- and *Y*-axis behavior could be performed. The cross-section dimensions of the specimen for the three-point bending tests were increased relative to standard dimensions in order to increase the number of cell rows arranged across the width of the specimen. The length of the specimen was correlated with the cross-section. 

No peripheral contours or upper/lower layers were generated, as their dimensions introduce additional variables into the analysis. In the reference system attached to the three types of structures, the *X*-axis defines the length, and the *Y*- and *Z*-axes define the cross-section. 

**Figure 6:**
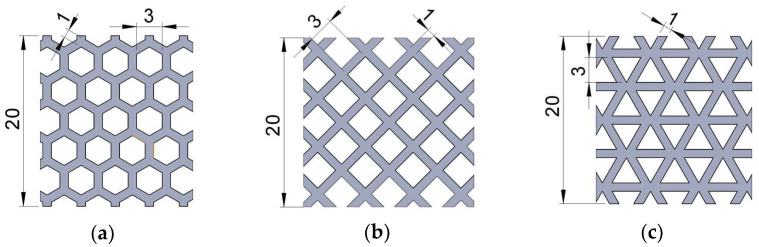
The detailed views of structures’ cells: (**a**) hexagonal, (**b**) grid, (**c**) triangles.

The structures defined by 2D patterns with regular polygonal cells (hexagon, square, triangle) are characterized by the specific dimensions of the polygons and the distances between two neighboring polygons (wall thickness) [38]. In this research the cell wall thicknesses were identical in all three structures, and the characteristic dimensions of the polygonal cells were chosen assuming that the specimen volumes were of the same scale order. A specific dimension of 3 mm was used to define the inner of the cells and the wall thickness was kept constant at 1 mm (Figure 6). The layout of the hexagonal/ grid/ triangular cells was made so that the structures were symmetrical relative to the median planes. Therefore, the *X*, *Y* and *Z* axes were the main axes of inertia.

To compare the mechanical behavior of the three structures, the differences in actual volume (reflecting material consumption) were calculated, based on the 3D models from SolidWorks (Table 1). The volume of the grid structure is 1.82% smaller compared to the hexagonal structure, while the volume of the triangular structure is 23.96% higher.

The 3D printing was done by fused filament fabrication (FFF), using an Ultimaker 2+ Connect printer (Ultimaker B.V., Utrecht, Netherlands). The geometries generated in SolidWorks were imported as stl file into the Ultimaker Cura 5.0.0 slicer software. The material used was Ultimaker Silver PLA (Ultimaker B.V., Utrecht, Netherlands) with a diameter of 2.85 mm. The main properties of the material (according to the manufacturer’s specifications) were: density—1.24 g/cm^3^, melting point—145–160 °C, thermal decomposition—250 °C, tensile modulus of elasticity—2890 MPa up to 3393 MPa, tensile stress at break—30.3 MPa up to 57.5 MPa, flexural modulus of elasticity—2693 MPa up to 3106 MPa and flexural strength—52 MPa up to 101.3 MPa. No pre-process treatments were applied to the filament. The print parameters (Table 2) were maintained constant for all three structures. 

The selection of the printing parameters was done in accordance with the manufacturer’s recommendations and previous research [16,39]. The specimens were placed in the center of the build plate, following the XY orientation. No support material was used. Each specimen was printed individually.

**Figure 7:**
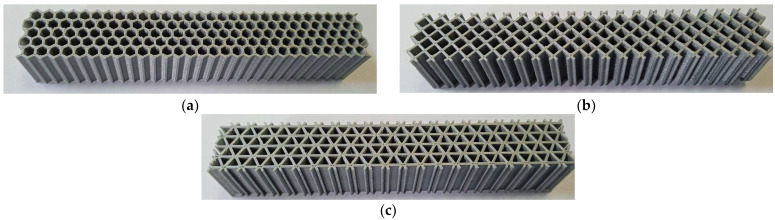
The test specimens: (**a**) hexagonal structure, (**b**) grid structure, (**c**) triangle structure.

Figure 7 shows the aspect of the three types of test specimens. No post-processing treatments were applied after 3D printing.

Two testing phases were completed to investigate the symmetric and asymmetric bending behavior of the structures:

(a) The analysis of the three-point bending behavior (symmetric bending);
(b) The analysis of the asymmetric bending behavior for cantilever beam loaded by a force disposed inclined to the main axes of inertia *Y* and *Z*.

**Figure 8:**
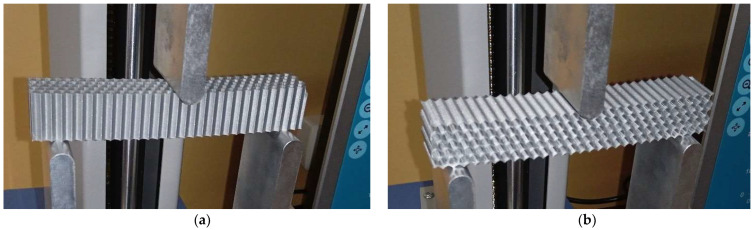

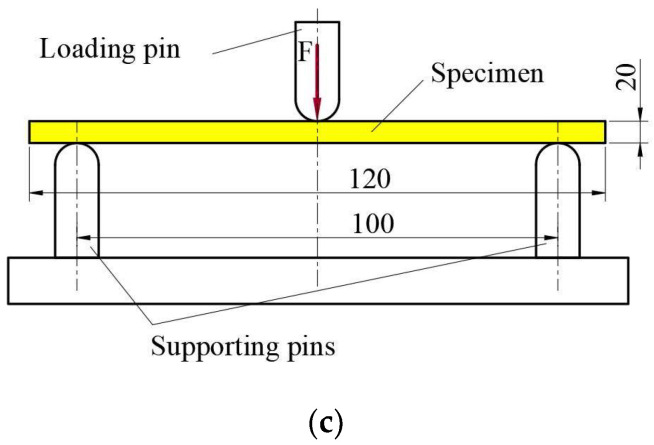
The 3-point bending setup for the hexagonal cells specimen: (**a**) force along the Y-axis of the specimen, (**b**) force along the Z-axis of the specimen, (**c**) device layout.

**Figure 9:**
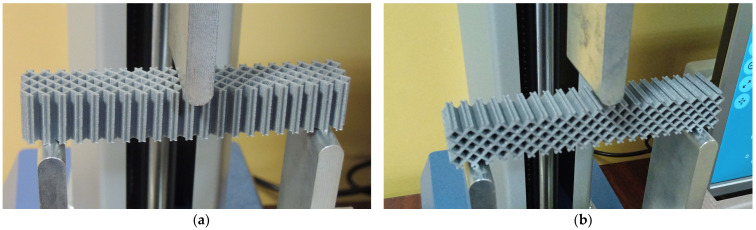
The 3-point bending setup for the grid cells specimen: (**a**) force along the Y-axis of the specimen, (**b**) force along the Z-axis of the specimen.

**Figure 10:**
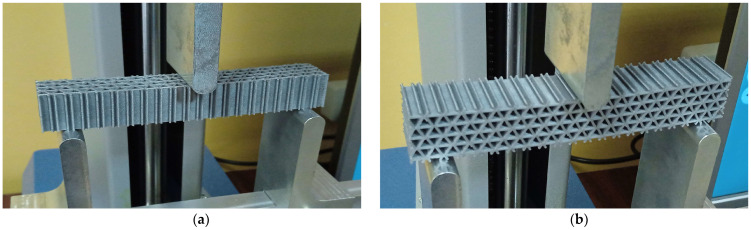
The 3-point bending setup for the triangle cell specimen: (**a**) force along the Y-axis of the specimen, (**b**) force along the Z-axis of the specimen.

The experimental three-point bending tests were carried out for two loading cases: force applied along the Y principal axis of inertia and force applied along the Z principal axis of inertia (Figure 8, Figure 9 and Figure 10). The tests were performed on a Mecmesin MultiTest 2.5 dV (PPT Group UK Ltd., Slinfold, UK) mechanical testing machine. The span between the 3-point bending device supporting pins was 100 mm and the force was applied in the middle section (Figure 8c). Displacements were measured for seven force values: 10 N, 20 N, 30 N, 40 N, 50 N, 60 N and 70 N. For the Z-direction force analysis, the specimens were positioned rotated (with the axis of the prismatic cells placed horizontally—Figure 8b, Figure 9b and Figure 10b). Three experimental tests were performed for each type of structure. The results are presented as force-deflection variations. Assuming that the force-deflection variations are linear (as the deflections are obtained in the elastic region of the material), the tangents of these variations were calculated.

**Figure 11:**
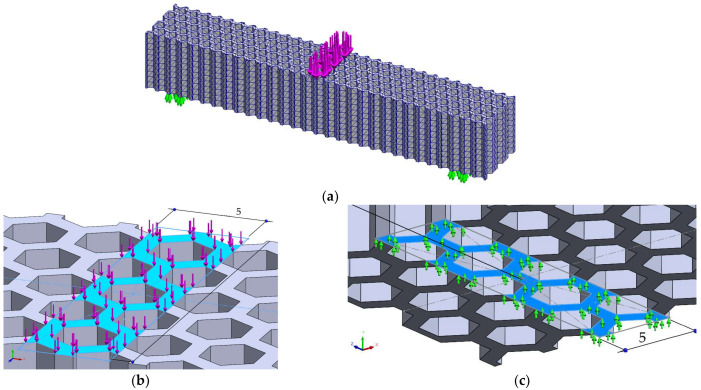
The FEM analysis setup for 3-point bending of the hexagonal structure—force applied along the *Y*-axis of the specimen: (**a**) general view, (**b**) detailed view of the loading force, (**c**) detailed view on fixing conditions (identically for both fixing areas).

**Figure 12:**
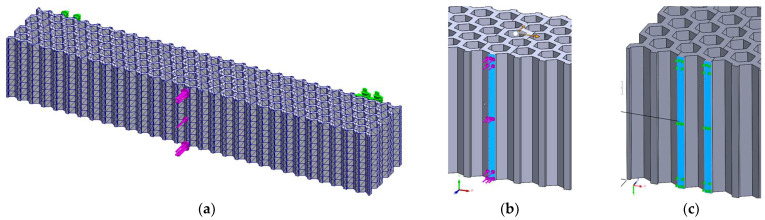
The FEM analysis setup for 3-point bending of the hexagonal structure—force applied along the *Z*-axis of the specimen: (**a**) general view, (**b**) detailed view of the loading force, (**c**) detailed view on fixing conditions (identically for both fixing areas).

Finite element analysis of the 3-point bending deflections was performed for loading layouts similar to the experimental tests (Figure 11 and Figure 12—example for the hexagonal structure; similarly loading layouts were used for the grid and the triangle structures). The force was varied from 10 N to 70 N, determining the force-deflection variations and the tangents of these variations. The set-up of the mesh was based on the convergence of the Y-axis maximum displacements. Subsequently, the mesh parameters were maintained identical for both bending cases of the same structure. Furthermore, the overall size of the finite elements was set to the same value (2 mm) for the three structures, resulting in: 52,444 finite elements with 95,468 nodes for the hexagonal structure, 50,663 finite elements with 95,739 nodes for the grid structure and 52,115 finite elements with 98,174 nodes for the triangle structure. 

The deviations between the tangents of the force-deflection variations obtained experimentally and those obtained by finite element analysis were analyzed. After the validation of the FEM simulations for 3-point bending, the analysis models for asymmetric bending were settled. Asymmetric bending assumes that the resultant force acts along an axis inclined to the principal axes of inertia *Y* and *Z*. 

**Figure 13:**
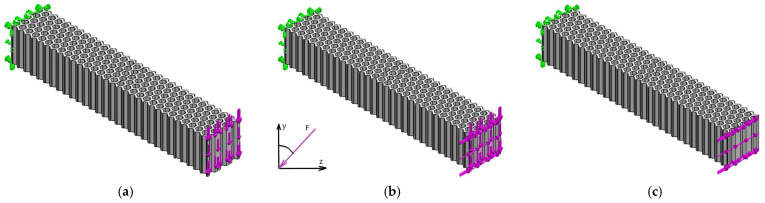
The FEM analysis setup for asymmetric bending of the hexagonal structure: (**a**) α = 0°, (**b**) α = 45°, (**c**) α = 90°.

In three-point bending the variation of the force direction implies the change of the fixing surfaces, which leads to additional variables introduced into the analysis. Therefore, the cantilever beam setup was used for the analysis of the displacement variation in asymmetric bending. The fixing conditions were applied on the left cross-section and the force was applied on the right cross-section of the beam for all loading cases (Figure 13—illustration for the hexagonal structure). An angle α was used to define the direction of the force in the (YZ) plane, relative to the *Y*-axis (Figure 13b). For α = 0° the force is applied along the *Y*-axis (Figure 13a) and for α = 90° the force is applied along the *Z*-axis (Figure 13c). The value of the applied force was kept constant at F = 5 N and the direction of the force was varied by 5° in the range 0°–90° (19 directions analyzed for each structure). In SolidWorks, the inclined force was applied as force projections (Figure 13b). For each type of structure, the variation of the displacement (Ures) versus angle α was plotted.

## 3. Results and Discussion

This chapter presents the results obtained in the two stages of the analysis: three-point bending along the *Y*- and *Z*-axes and asymmetric bending of a cantilever beam.

### 3.1. Three-Point Bending

The three-point bending layout corresponds to a simply supported beam with force applied in the middle section. In this case, the maximum deflection $U_{3\mathrm{PB}\;\max}$ of a beam with constant cross-section can be calculated with Equation (1):

(1) $$U_{3\mathrm{PB}\;\max}=\frac{F\cdot l^{3}}{E\cdot I}$$

where l is the beam length (the length between the two supporting pins of the three-point bending device), E is Young’s modulus and I is the axial moment of inertia (Iz at *Y*-axis loading, respectively Iy at *Z*-axis loading).

For a square cross-section the moments of inertia along the *Y* and *Z* axes are equal Iz/Iy=1, resulting in equal deflections on bending along the two axes Uy=Uz. For the structures with a cellular infill, the axial moments of inertia vary with the position of the cross-section along the *X*-axis. In addition, the axial moments of inertia Iz and Iy in any cross-section are not equal to each other and the ratio Iz/Iy depends on the position of the cross-section. The ratio of maximum deflection Uy/Uz will thus be dependent on the variation of the Iz/Iy ratios.

The experimental tests carried out at three-point bending aimed to determine the ratios of the maximum deflections Uy/Uz and to calculate the tangents of the force-deflection variations (these tangents reveal the elastic behavior of the structures).

**Figure 14:**
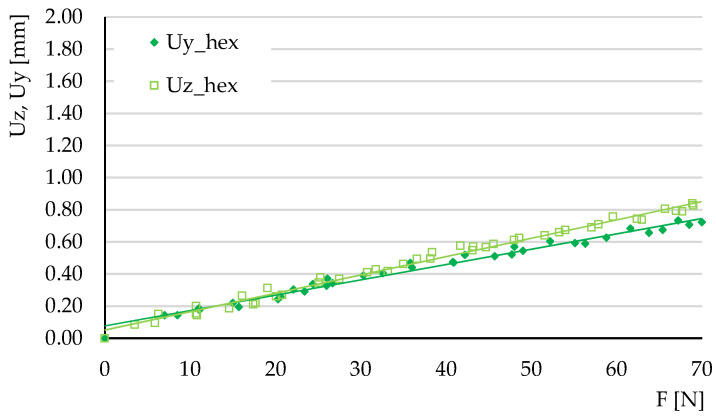
The variations of deflections *Uy* = *f*(*Fy*) and *Uz* = *f*(*Fz*) for the hexagonal structure, in experimental three-point bending.

**Figure 15:**
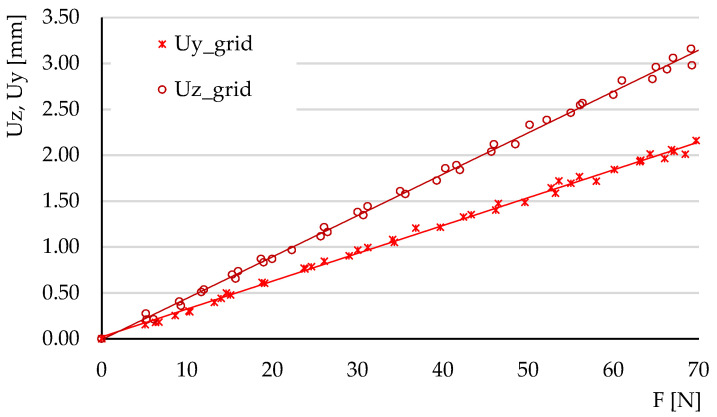
The variations of deflections *Uy* = *f*(*Fy*) and *Uz* = *f*(*Fz*) for the grid structure, in experimental three-point bending.

**Figure 16:**
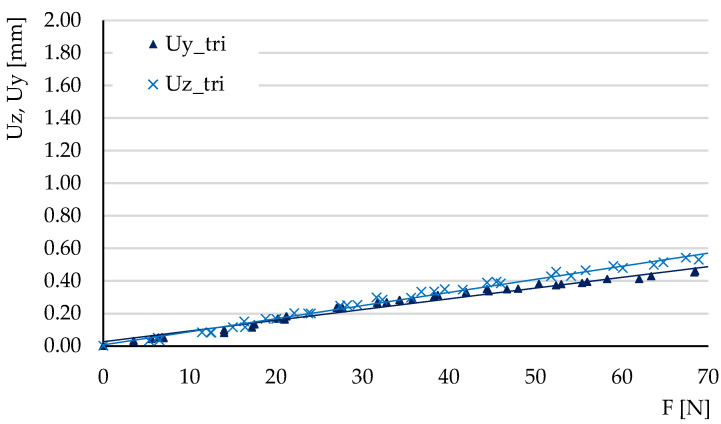
The variations of deflections *Uy* = *f*(*Fy*) and *Uz* = *f*(*Fz*) for the triangle structure, in experimental three-point bending.

The deflections obtained in the three tests carried out for each type of structure were represented graphically, plotting the average force-deflection variation. The tests were carried out in the elastic domain; thus, the variations were linear. Figure 14, Figure 15 and Figure 16 show the deflection variations Uy=f(Fy) (force applied on the *Y*-axis of the specimen) and Uz=f(Fz) (force applied on the Z-direction) resulting from the experimental tests for the three types of structures. For the hexagonal structure the variations Uy=f(Fy) and Uz=f(Fz) have similar slopes. The same type of behavior is observed for the triangle structure. In the grid structure the tangent of the variation Uz=f(Fz) is substantially higher than the tangent of the variation Uy=f(Fy).

**Figure 17:**
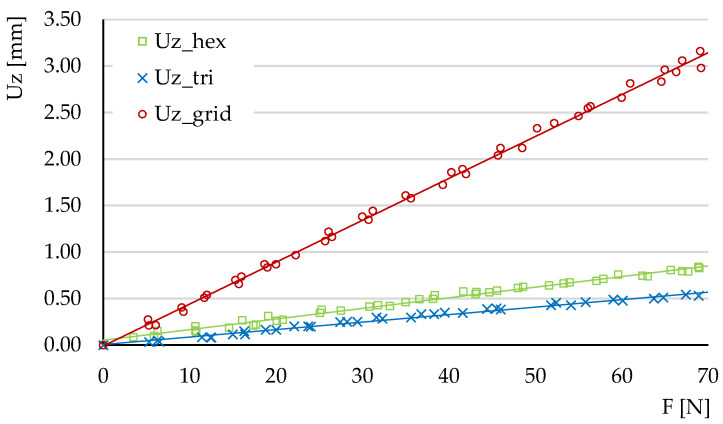
The comparative analysis of the variation of deflection Uz=f(Fz) for the hexagonal, triangle and grid structures, in experimental three-point bending.

Figure 17 shows a comparison of the variations Uz=f(Fz) for the three types of structure. It can be seen that the triangular structure has the smallest deflections, followed by the hexagonal structure. The differences between the deflections of these two types of structure and the deflections of the hexagonal structure are large.

The comparative analysis must also include the volume of each structure. As shown in Table 1, hexagonal cell structures and grid cell structures have similar volumes $V_{grid}/V_{hex}=0.98$. The ratio of the tangents of the deflection functions  θz=tan(Uz=f(Fz)) is $\theta z_{grid}/\theta z_{hex}=3.94$. Thereby, at the same material consumption, the hexagonal structure has smaller deflections compared to the grid structure.

For the comparative analysis between the hexagonal structure and the triangle structure the volume ratio is $V_{tri}/V_{hex}=1.23$, while the ratio of the tangents of the deflection functions is $\theta z_{tri}/\theta z_{hex}=0.70$. It follows that a 23% higher material consumption in the triangle structure leads to a 30% decrease of deflection compared to the hexagonal structure.

**Figure 18:**
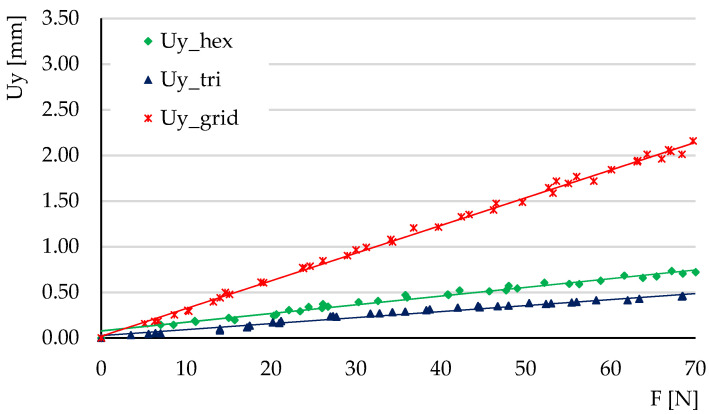
The comparative analysis of the variation of deflection Uy=f(Fy) for the hexagonal, triangle and grid structures, in experimental three-point bending.

Figure 18 shows a comparison of the variations Uy=f(Fy) for the three types of structures. The tangent ratios of the deflection variations θy=tan(Uy=f(Fy)) are: $\theta y_{grid}/\theta y_{hex}=3.18$ and $\theta y_{tri}/\theta y_{hex}=0.69$. The tendencies are similar with the Uz=f(Fz) variations.

**Figure 19:**
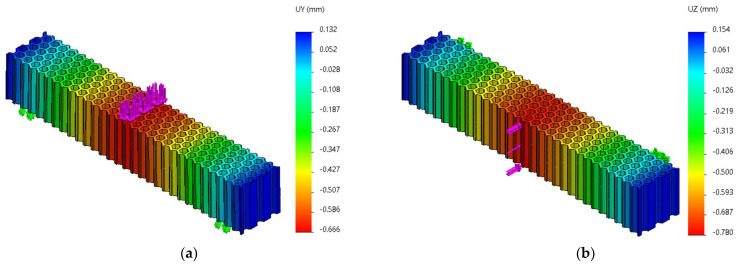
The *Uy* and *Uz* displacements charts obtained from FEM analysis of three-point bending of the hexagonal structure: (**a**) *Fy* = 70 N, (**b**) *Fz* = 70 N.

**Figure 20:**
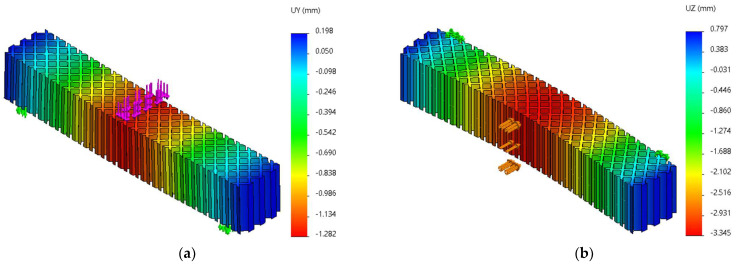
The *Uy* and *Uz* displacements charts obtained from FEM analysis of three-point bending of the grid structure: (**a**) *Fy* = 70 N, (**b**) *Fz* = 70 N.

**Figure 21:**
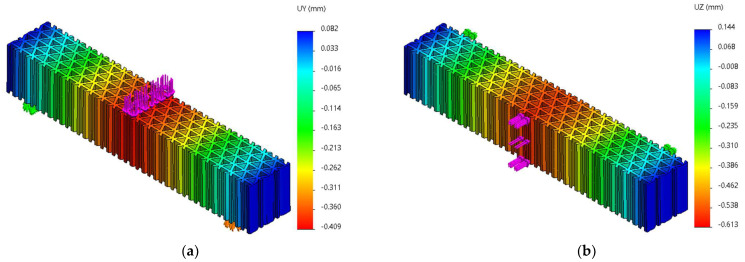
The *Uy* and *Uz* displacements charts obtained from FEM analysis of three-point bending of the triangle structure: (**a**) *Fy* = 70 N, (**b**) *Fz* = 70 N.

FEM analyses of three-point bending were performed for seven force values, ranging from 10 N to 70 N, determining the functions Uy=f(Fy) and Uz=f(Fz) and the tangents of these functions. Figure 19, Figure 20 and Figure 21 show the charts of displacements for F=70 N.

Table 3 summarizes the values of the tangents θz and θy obtained experimentally and by numerical simulation of three-point bending. The values of the coefficient of determination R^2^ are also presented. It is observed that for the variations determined by experimental measurements, the values of this coefficient are situated between 0.975 and 0.997, indicating small values of the deviations.

The conclusions of this analysis of the three-point bending displacements should be correlated with the orientation of the structures’ cells. The effect of changing the orientation of hexagonal/triangular/grid cells through rotation in the (XZ) plane was not included in this analysis. The cell orientation influences the consumption of the material [40] and the mechanical behavior of the structures [41]. Furthermore, changing the position of the force relative to the center of the polygonal cells determines the changes in the mechanical behavior of the structure [42].

### 3.2. The Variation of Resultant Displacement in Unsymmetrical Bending

For a cantilever beam loaded with asymmetric force *F*, oriented at angle *α* to the *y*-axis, the maximum resultant displacement can be calculated by the equation:

(2) $$U_{res}={\left({\left(\frac{F\cdot \cos\alpha \cdot l^{3}}{E\cdot I_{z}}\right)}^{2}+{\left(\frac{F\cdot \sin\alpha \cdot l^{3}}{E\cdot I_{y}}\right)}^{2}\right)}^{1/2}$$

where: l is the length of the bar; E is Young’s modulus; and Iy and Iz are the axial moments of inertia. From Equation (2) it is observed that if Iz=Iy (e.g., square cross-section), then U_res is constant for any value of angle α.

For the three geometries considered here the cross-section is fitted into a 20 × 20 mm^2^ square, the *Y* and *Z* axes are the principal axes of inertia, but the axial moments of inertia Iy and Iz are not equal and are variable, depending on the position of the cross-section on the *X* axis.

**Figure 22:**
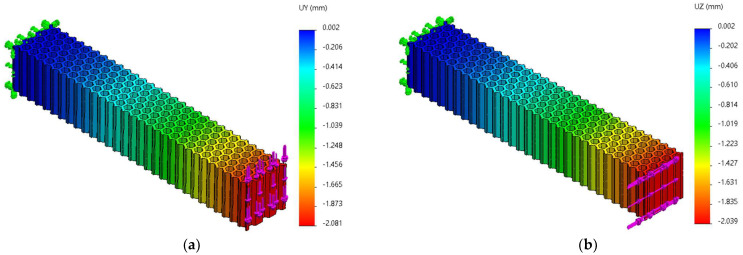
Cantilever beam. The displacement charts for the hexagonal structure: (**a**) α = 0°, (**b**) α = 90°.

**Figure 23:**
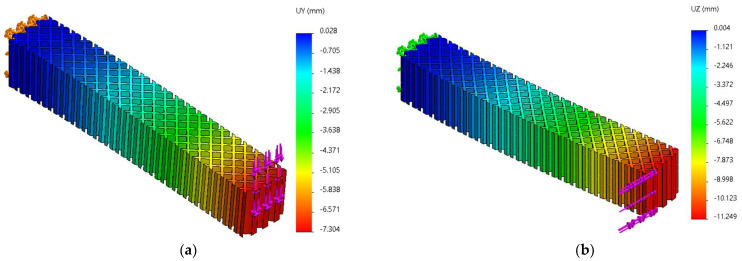
Cantilever beam. The displacement charts for the grid structure: (**a**) α = 0°, (**b**) α = 90°.

**Figure 24:**
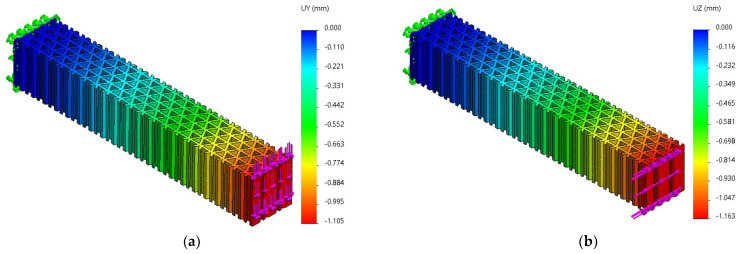
Cantilever beam. The displacement charts for the triangle structure: (**a**) α = 0°, (**b**) α = 90°.

To establish the variation of the displacements as a function of the orientation of the force (angle α), the value of the resultant force was kept constant and the angle α was varied by 5°, in the range 0°–90°. Figure 22, Figure 23 and Figure 24 show the distribution of displacements for the three structures at angles α = 0° and α = 90°. The similar values of the maximum displacements for the hexagonal structure are observed, with the ratio $U_{hex\left(\alpha =0\right)}/U_{hex\left(\alpha =90\right)}=1.02$. For the triangular cell structure, the displacement ratio is $U_{tri\left(\alpha =0\right)}/U_{tri\left(\alpha =90\right)}=0.95$. The grid cell structure shows a significant variation in the resulting displacement $U_{grid\left(\alpha =0\right)}/U_{grid\left(\alpha =90\right)}=0.64$ and larger displacements relative to the hexagonal and triangular structures.

**Figure 25:**
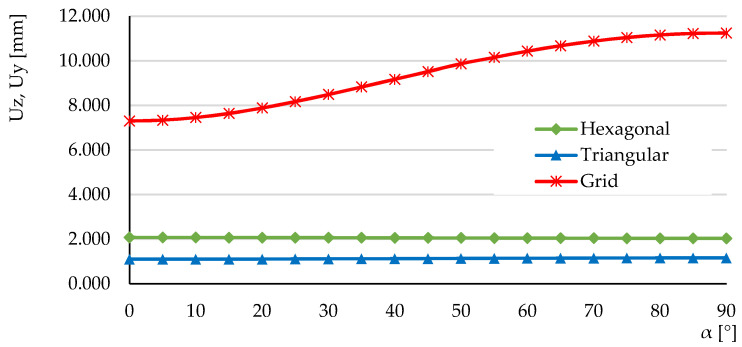
The influence of force orientation on the resultant displacement at asymmetric bending, Ures=f(α).

Figure 25 shows the variation of the resultant displacements as a function of the angle α of inclination of the force. It can be seen that the extreme values are obtained for α = 0° and α = 90°. For the grid structure the largest displacement is obtained for the orientation of the force along the *Z*-axis (α = 90°), a conclusion also drawn from the experimental analysis at three-point bending.

The low variation of the resultant displacement in hexagonal and triangular geometries shows that these structures can be used in beam-like mechanical components loaded with force of variable orientation.

The conclusions resulting from the analysis of the displacements under asymmetric bending should be correlated with the cell orientations used for the three types of structure. Changing the orientation of the cells by rotation in the (XZ) plane can cause the change of the position of the principal axes of inertia.

## 4. Conclusions

The use of cellular structures for the core of mechanical components leads to a reduction in mass and material consumption and may conduce, at the same time, to an optimized stress distribution and an improved elastic behavior. To achieve these advantages, the mechanical behavior of these structures, as well as the variation of the mechanical properties in relation to the main axes of inertia, has to be known. 

The number and thickness of top/bottom layers and the number of peripheral contours constitute variables that influence the analysis of infill types, especially when using low infill densities or test specimens with a small cross-section area. An alternative is the testing of structures without shells, as presented in this paper.

The analysis of symmetric and asymmetric bending displacements for the three structures considered in this paper (hexagonal structure, grid structure and triangular structure) led to the following conclusions:

- The grid cell structure has significantly larger displacements than the hexagonal cell structure at the same material consumption.
- At the same material consumption, the triangular cell structure has slightly smaller displacements than the hexagonal cell structure.
- For hexagonal/triangular cell structures the displacements do not vary significantly with the orientation of the force relative to the principal axes of inertia. These types of structure are suitable for mechanical components where the force changes direction in the YZ plane.
- For the grid structure the variation of displacements with the orientation of the force relative to the principal axes of inertia is large; displacements are higher when the force acts perpendicular to the axis of the prismatic cells of the grid geometry.

The analyzed structures show symmetry with respect to the median planes. The rotation of the cells in the XZ plane can cause the change of the principal axes of inertia and the modification of the bending behavior. These issues will be analyzed in future research.

## Figures and Tables

**Table 1:** The comparative analysis of volume for the three specimens.

Sample	Volume [mm^3^]	Volume Variation rel. to Hexagonal [%]
Hexagonal	21,353.07	0.00
Grid	20,964.76	−1.82
Triangular	26,468.87	23.96

**Table 2:** The 3D printing parameters.

Parameters	Values
Process	fused filament fabrication (FFF)
Layer thickness, t	0.1 mm
Printing head temperature, T_H_	210 °C
Build plate temperature, T_B_	60 °C
Printing speed, s_p_	50 mm/s
Nozzle diameter, d_n_	0.40 mm
Filament diameter, d_f_	2.85 mm
Build orientation (acc. to [15])	XY
Material	Ultimaker PLA Silver

**Table 3:** The comparative analysis the tangents of the Uy=f(Fy) and Uz=f(Fz) functions obtained by experiment and the FEM simulation of three-point bending.

Geometry	*Uy* = *f*(*Fy*)				*Uz* = *f*(*Fz*)			
	Experimental		FEM		Experimental		FEM	
	θy	R^2^	θy	R^2^	θz	R^2^	θz	**R^2^**
Hexagonal	0.0095	0.982	0.0095	1	0.0114	0.989	0.0113	1
Grid	0.0303	0.997	0.0183	1	0.0450	0.997	0.0478	1
Triangular	0.0066	0.975	0.0058	1	0.0080	0.987	0.0088	1

## Data Availability

The data presented in this study are available on request from the corresponding author.
